# Assessment of tricalcium phosphate/titanium dioxide (TCP/TiO2) nanocomposite scaffold compared with bone autograft and hydroxyapatite (HA) on the healing of segmental femur bone defect in rabbits

**DOI:** 10.1007/s10856-022-06694-z

**Published:** 2022-12-08

**Authors:** Hoseyn Sonbolekar, Jahandideh Alireza, Asghary Ahmad, Saeed Hesaraki, Abolfazl Akbarzadeh

**Affiliations:** 1 Department of Clinical Science, Science and Research Branch, Islamic Azad University, Tehran, Iran; 2 Department of Pathobiology, Science and Research Branch, Islamic Azad University, Tehran, Iran; 3 Drug Applied Research Center, Tabriz University of Medical Sciences, Tabriz, Iran; 4 Universal Scientific Education and Research Network (USERN), Tabriz, Iran

## Abstract

Bone healing is a tissue process after a surgical operation. Many formulated materials have been designed for improving these procedures. The purpose of this study was to evaluate the effectiveness of nanocomposite tricalcium phosphate scaffolds combined with Titanium dioxide scaffold (TCP/TiO2) for femoral defects regeneration in rabbits. We studied 80 mature male New Zealand white rabbits weighing between 3 and 3.5 kg. Rabbits were subdivided into four groups. Anesthesia was performed before surgical operation by 50 mg/kg Ketamine 10% and 5 mg/kg xylazine 5% intramuscularly. We inducted a 6 × 5 mm diameter cylinder defect on the femur. Animals were separated into four trial groups of 20 animals each. After defecting, the experimental groups include control, autograft, hydroxyapatite, and TCP/TiO2 (received pure nanocomposite TCP/TiO2 material). A pathologist evaluated the sections on days 15, 30, 45, and 60 after surgery. The improvement of new and lamellar bone formation was the best in the nanocomposite TCP/TiO2 group at various point times, especially 60 days after surgery. We found that TCP/TiO2 nanocomposite has a significant improving function in the remodeling of bone in the defect areas.

**Figure:**
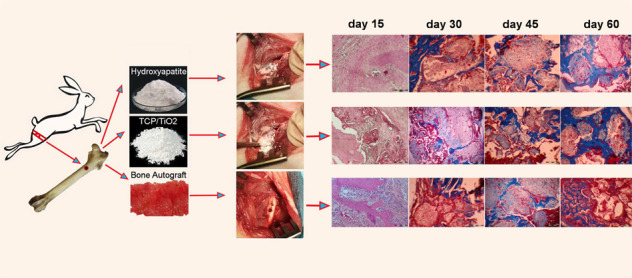
Graphical abstract

Graphical abstract

## Introduction

The ceramides are hydroxyapatite, Tricalcium phosphate (TCP), and calcium sulfate, which can improve bone defects. Bone grafts are categorized into autograft, allograft, xenograft, ceramics, and growth factors. The growth factors include demineralized bone matrix, Platelet-Rich Plasma, and Bone morphogenetic proteins. The bone substitute materials are essential to regular surgery in orthopedics and dentistry [1]. The autogenous broken bone fragments from different sites are the best way to fill fracture defects. This task is due to its ability to provoke bone synthesis. The preparation of bone particles from various areas of the patient’s body is associated with bone necrosis, nerve injury, new surgical wounds, and the resulting pain [2, 3]. Structural substrates such as nanocomposite TCP and collagen can lead to the better bone formation by creating conditions similar to autograft broken bone fragments [4]. Today, non-cellular bone fragments obtained from animals are xenografts used as substructures to fill bone defects due to their high similarity in bone structure and bone lamella and trabecula [2, 4]. Various animal bone xenografts can induce proper bone formation due to providing a well-formed bony environment [5]. The xenogeneic bone is used for extremities fracture surgery, providing a long-term scaffold for successful bone remodeling [5]. Considerable evidence in bone surgery revealed a variety of xenografts available in various forms of bony granules to bone glues [6]. The viscosity of glues causes the filling of irregular borders of bone defects. This viscosity allows an increased approach that aids clinicians to overwhelming implantation missteps [7]. The synthesized bone glues comprise calcium phosphate-containing bone framework granules with a hyaluronic or collagen hydrogel. It has a water retention capacity [7, 8]. It has been proved that more proteins in the extracellular matrix can enable better bone healing by increasing chemotaxis and migration of osteoblast and endothelial cells [9–11]. Moreover, these stromal molecules can proliferate endothelial cells and osteogenic stem cells [12]. Some research revealed that using collagen and hydroxyapatite could increase osseous healing [4]. The immune and inflammatory response associated with bony form materials has received control against inflammation that can induce better bone healing in the defects [13, 14]. In the following, some investigators proved that hydroxyapatite or similar biomaterials could change the M1 macrophages to M2-form, which is assumed to decrease the inflammatory response against foreign body response and promote better bone remodeling [15]. TCP nanocomposite in use with collagen can fill the bone defects. Then its granules can be used to scaffold the bone fractures [4]. The half-life of the hydroxyapatite molecule in the local defect is about 36 h. Thus, it is necessary to design a better combination and composite with a low quantity of implant material and a higher half-life preserved in the defect. Nanocomposite biomaterials allow devising particular characteristics to control cooperation between polymers and nanoparticles better. Polymer nanocomposite biomaterials possess better engineering characteristics when compared with the other types of composites [4, 11]. This study aimed to assess new bone healing of defects by TCP/TiO2 nanocomposite scaffolds. Titanium causes a decrease in the wettability of composite. Titanium container particles can increase the deposition and hardness of phosphate calcium [11].

We did not see the use of tricalcium phosphate/titanium dioxide (TCP/TiO2) nanocomposite in any article. This composition consists of components, some of which may have a long history of research, such as tricalcium phosphate. However, titanium oxide is limited in text studies, and the nanocomposite form has never been researched.

## Material and methods

### Synthesis of TiO2 nanoparticles and tricalcium phosphate/titanium dioxide (TCP/TiO2) nanocomposite

Titanium Tetra Isopropoxide (TTIP, C12H28O4Ti, 97%), Ethanol (C2H5OH, 96%), was purchased from Merck. Tricalcium phosphate was purchased from Sigma–Aldrich. Firstly, 0.05 M of titanium tetra isopropoxide is dissolved in 10 ml of ethanol solution under continuous stirring for 20 min. Subsequently that, add a limited drops of distilled water to form the dispersion medium. The product was placed on the ultrasonic bath for 15 min and then, the solution was transferred into an autoclave at 150 °C for 5 h. The filtered sample was dried oven at 130 °C for 7 h, and at 500 °C for 1 h. The TiO2 NPs was collected [16]. Tricalcium phosphate nanocomposites dispersed with titanium oxide were produced by using powder of nanostructured hydroxyapatite bone matrix, formerly obtained by the dissolution-precipitation technique.

### In vivo study

We designated the 80 mature male New Zealand white rabbits, 6–8 months of age and 2.5–3 kg weight, and randomly allocated them into four different research groups. Thus, each group consisted of three animals, including 20 per study time point on 15, 30, 48, and 60 days. We continued our research after concerning ethical permission from the Tehran Science and Research Committee number 5393. The experimental group includes control: defect-bearing surgery without any treatment. Autograft: treated by autograft bone particles from the defecting area. HA: The defects were implanted with hydroxyapatite. TCP/TiO2: The defects were implanted with nanocomposite TCP/TiO2 granules. Penicillin G procaine (40,000 IU/kg IM, twice a day) and dexamethasone (0.6 mg/kg, IM) were administered three days after surgery. Experimental animals were kept in separate cages to prevent self-injury (Table 1).

**Table 1:** Overview of the experimental animals per group and time point

	Control	Autograft	HA	TCP/TiO_2_
15 days	5	5	5	5
30 days	5	5	5	5
45 days	5	5	5	5
60 days	5	5	5	5
Number per study group	20	20	20	20
Total number	80 experimental animals			

### Surgery procedure

A 5 cm long incision was performed in the aseptic condition along the medial right upper hind limb and the mid diaphyseal surface of the femur. The periosteum was removed from the diaphysis by a periosteal elevator. Furthermore, we perform a 6 × 5 mm diameter cylinder defect in the femur. We washed the surgery site with normal saline and put the preserved periosteum on the defect site. Then we covered it with the overlying muscles. The surgery site was treated according to the cure protocol for each rabbit [4]. After surgery, we checked the site of the defects daily to prevent infection or hemorrhagic condition.

### Sample preparation and staining procedures

Twenty rabbits were euthanized through euthasol (400 mg/ml) at each time point on days 15, 30, 45, and 60 post-surgery. We operated histological workup following removing the implantation area. We fixed the defected femur segment and explanted substitutes in the buffered 10% formalin solution for seven days. The Femoral explants contained both bone defect ends. The selected osseous tissues were put in the acid for decalcification. The sections were stained with routine H&E and azan Mallory trichrome (detecting fibrosis) and analyzed under a light microscope. We continued the other histological processing, including automatic dehydration, clearing by xylene, paraffin embedding, and sectioning. Sections with a thickness of 6 µm were prepared using Leica microtome (semi-automatic 2245, Germany). The sections were stained with routine H&E and azan Mallory trichrome (detecting fibrosis) and analyzed under a light microscope.

### Histopathological

To evaluate the inflammatory tissue response and osseous formation and integration, we evaluated histopathology (Table 2) based on Allen’s scoring system [17]. Thereby, we compared TCP+TiO2 and autograft (control group) to evaluate the effect of the Nano TiO2. Histological lesions were graded based on the osseointegration and the types of inflammatory response [4].

**Table 2:** Lane and Sandhu histopathological scoring system modified by Heiple et al. [21]

Union (proximal and distal evaluated separately)	
No evidence of union	0
Fibrous union	1
Osteochondral union	2
Bone union	3
Complete reorganization of shaft	4
Cancellous (spongy) bone	
No osseous cellular activity	0
Early apposition of new bone	1
Active apposition of new bone	2
Reorganizing cancellous bone	3
Complete reorganization of cancellous bone	4
Cortical bone	
Non	0
Early appearance	1
Formation under way	2
Mostly reorganized	2
Completely formed	10
Marrow	
None is resected area	0
Beginning to appear	1
Present in more than half of the defect	2
Complete colonization by red marrow	3
Mature fatty marrow	4
Total points possible per category	
Proximal union	4
Distal union	4
Cancellous bone	4
Cortex	4
Marrow	4
Maximum score	20

### Statistical analysis

Two-way ANOVA, Dunnett posthoc test was used for statistical analysis via the GraphPad Prism 9.0 software. The data was semi-quantitative Based on histopathology. The inter-individual consequences were designated as significant if the p-values were less than 0.05 (*p ≤ 0.05), less than 0.01 (**p ≤ 0.01), and less than 0.001 (***p ≤ 0.001). Finally, the data were presented as mean and standard deviations.

## Results

### Descriptive statics of healing phenomena

#### Descriptive statics of the union (both proximal and distal) phenomena

**Fig. 1:**
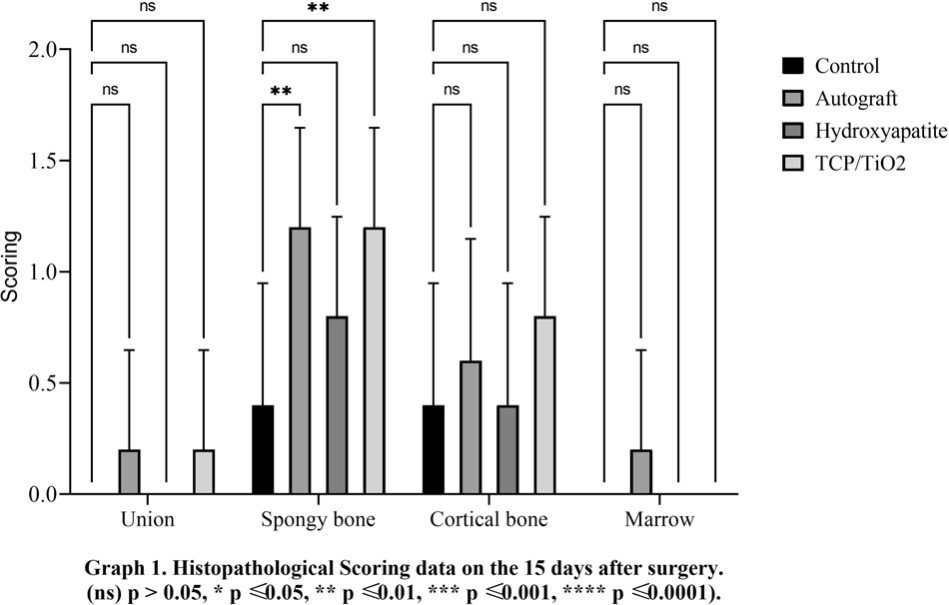
Histopathological scoring data on the 15 days after surgery. (ns) *p* > 0.05, *p ≤ 0.05, **p ≤ 0.01, ***p ≤ 0.001, ****p ≤ 0.0001

**Fig. 2:**
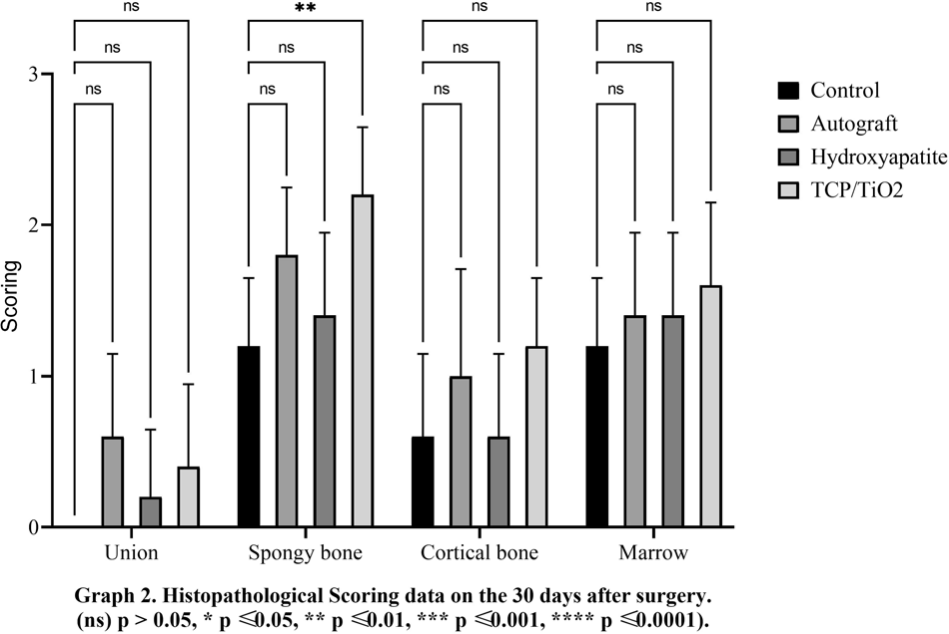
Histopathological scoring data on the 30 days after surgery. (ns) *p* > 0.05, *p ≤ 0.05, **p ≤ 0.01, ***p ≤ 0.001, ****p ≤ 0.0001

**Fig. 3:**
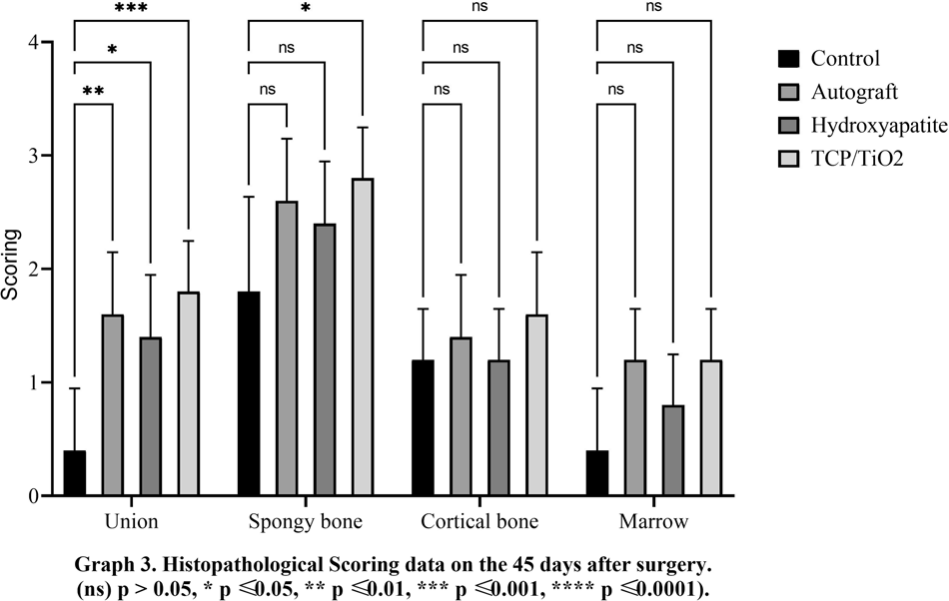
Histopathological scoring data on the 45 days after surgery. (ns) *p* > 0.05, *p ≤ 0.05, **p ≤ 0.01, ***p ≤ 0.001, ****p ≤ 0.0001

**Fig. 4:**
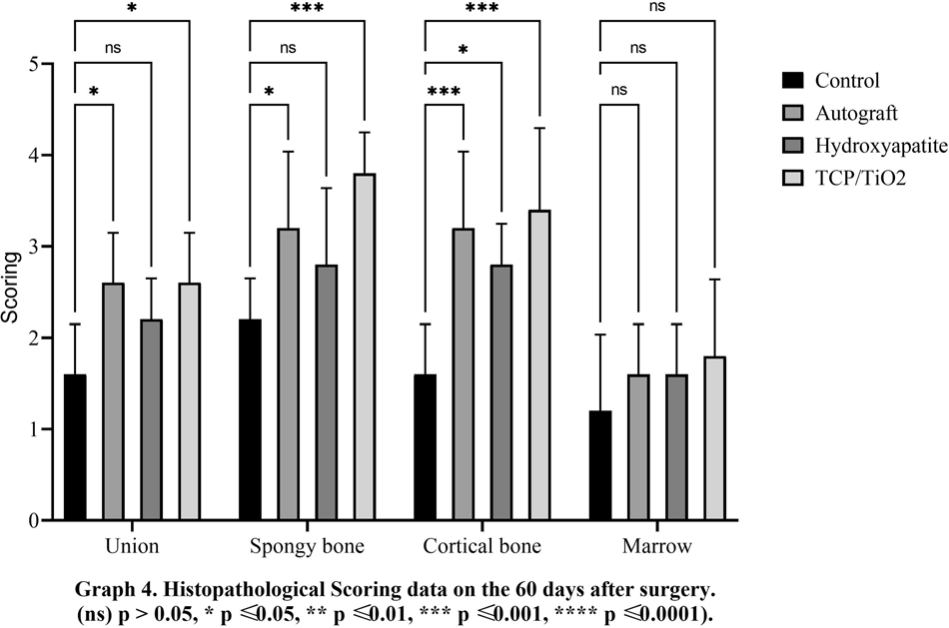
Histopathological scoring data on the 60 days after surgery. (ns) *p* > 0.05, *p ≤ 0.05, **p ≤ 0.01, ***p ≤ 0.001, ****p ≤ 0.0001

There was a minute developing primary bone in all treating groups from the edges of the defects on day 15 postoperative. Logically, the union was not supposed to be observed at this point time. Data on day 30 revealed no significant difference between all experimental groups (*p* < 0.05). The best speed of bone union formation, at day 45, belonged to the nanocomposite TCP-TiO2 treated group. The autograft group improved better compared to the control and HA-treated groups (*p* < 0.05). Interestingly, the significant highest score on day 60 belonged to the autograft in addition to the nanocomposite TCP-TiO2 treated group similarly, and the worst healing belonged to the control group (Figs. 1–4).

#### Descriptive statics of the spongy bone phenomena

The autograft and TCP-TiO2 treated groups initiated primary bone formation sooner than the others in the spongiosa index, 15 days after the surgery (*p* < 0.05). The TCP-TiO2 group produced more new primary bone trabeculae than the others 30 and 45 days after surgery (*p* < 0.05). On the 60th day, the best bone lamella improvement belonged to the nanocomposite TCP-TiO2 treated group, and the control and HA-treated groups were both lower. Albeit, the autograft was among the others, not as improved as the TCP-TiO2 group (Figs. 1–4).

#### Descriptive statics of the cortical bone phenomena

The cortical bone surrounding the defect area did not regenerate any of the lost compact bone in all groups of 15, 30, and 45 days after surgery. On the 60th day, concentric semi-arched lamellae began to form in the autograft, TCP-TiO2, and HA groups. There was a significant difference between the autograft, TCP-TiO2, and HA, too (*p* < 0.05) (Figs. 1–4).

#### Descriptive statics of the marrow phenomena

On day 15, the marrow formation of all groups was not significant. On day 30 after surgery, the control group has the granulation tissue in the space in the defect area. The healing process and marrow regeneration were nonsignificantly in the nanocomposite TCP-TiO2 treated group. By day 45 of the healing process, the highest score belonged to the nanocomposite TCP-TiO2 treated and the autograft groups more than the HA and control groups. On the 60th day, there were not any significant differences in the presence of the marrow (*p* < 0.05) (Figs. 1–4).

### Results of the other histological finding

**Fig. 5:**
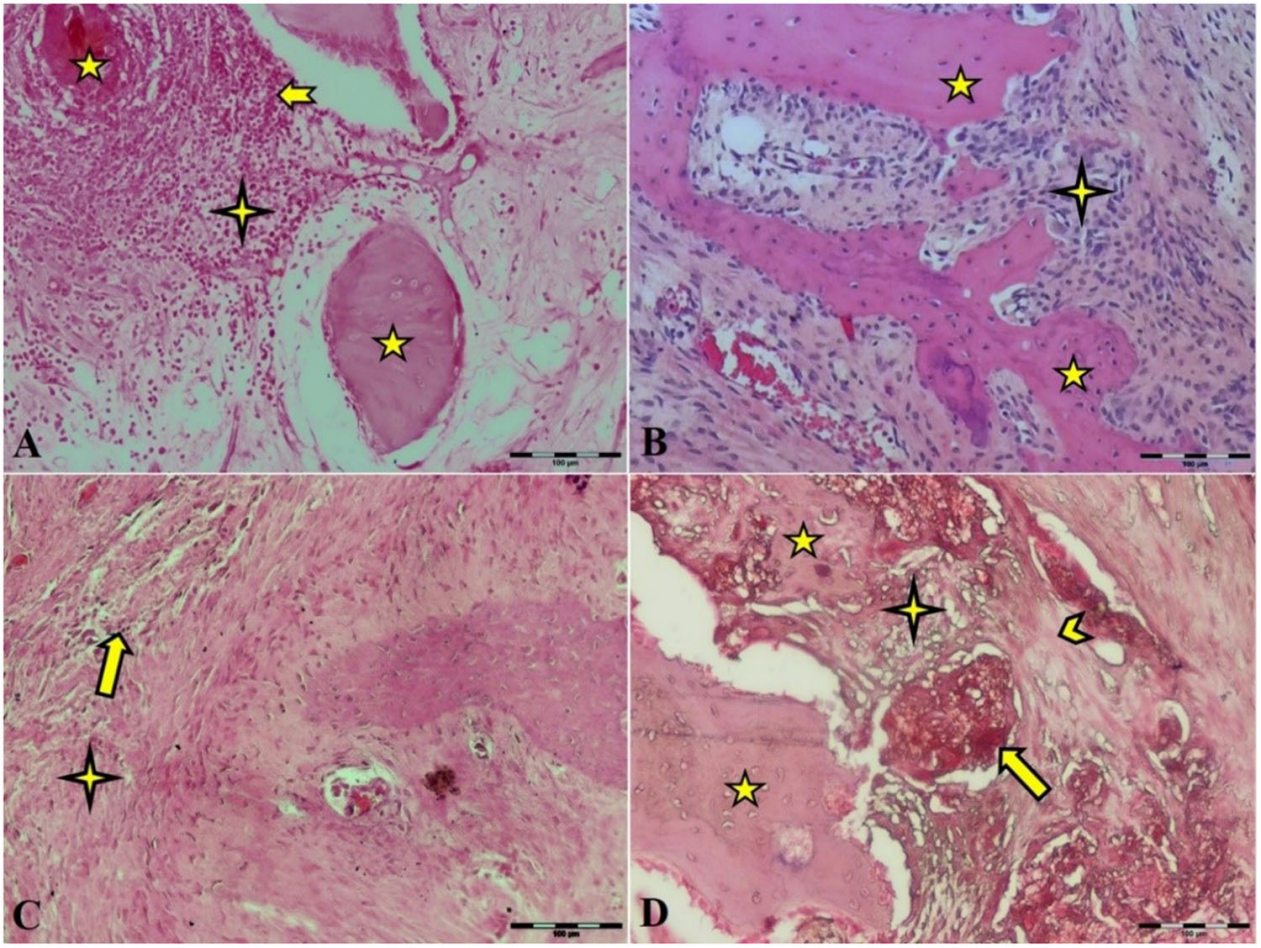
Microscopic section from the healing site of defect on the 15th day postoperation. **A** Control group has abundant leukocyte infiltration in granulation tissue. **B** The autograft group shows bone remains. **C** HA and **D** TCP-TiO2 materials in the center of the defect. Scant new bone formation is shown in the TCP-TiO2 group (HE × 200). star: remain previous bone; notched arrow: leukocyte infiltration; arrow: substitute material; chevron: new bone; 4-point star: granulation tissue

There was scantly local newly formed bone in the defective area of all treated groups at 15 days post-implantation. The new bone formation originated from the edges near the defects of the femur in the autograft and TCP-TiO_2_ treated groups that were not significant. There was more leukocyte infiltration in the control group than in the others (Fig. 5A–D).

At 30 days post-implantation, we showed the primary bone integrated into the substitutes. It may be due to the osteoconductive ability of both materials in the HA and TCP/TiO2 groups (Fig. 5A, B). Nevertheless, the materials had no efficient protrusion of the connective tissue (Fig. 5A, B). The overall inflammation was mainly composed of polymorphonuclear cells, lymphocytes, and macrophages. The inflammatory response has more mononuclear numbers in HA and TCP/TiO2 groups than in control and autograft groups on days 15 and 30 postoperation.

The rate of bone development was improved on day 45 compared to day 30 in all groups. This bone includes the scant local formation of the lamellar bone. In the TCP-TiO_2_ group, we fund more bone trabecular creeping to the material than the HA group or others. In addition, the inflammatory response rate was reduced compared with 30 days after postoperation.

At 60 days after implantation, the lamellar spongy bone had filled about half of the area that had been defected. This lamellar bone filled the inside parts of the defect area and integrated most of the materials in all autograft and material study groups (Fig. 5D, E). However, the bone-forming matrix infiltrated half of the material surfaces (Fig. 5D, E). Microvascular-rich connective tissue remained in the center of the defect areas (Fig. 5D, E). At these 60 days, the connective tissue filled about half of the defect space in the control group. However, the new bone formation was increased from the borders towards the central defect regions in the TCP-TiO_2_ group more than the others (Fig. 5F). There was a mild inflammatory response in all four-study groups.

## Discussion

Nanoengineering strategies have a specific electrospinning ability; develop a framework for medical surgery protocols. This strategy is accurate technology since it is a comparatively easy and economical process for creating nanocomposite materials useful for many biomedical purposes such as xenograft engineering [18]. Various implantation of materials in the fractured bone can improve regeneration, not only in medical orthopedics but also in veterinary counterparts. They are applied to improve more complicated conditions such as deeply irregular-shaped defects. Material glues more used as bone grafts have a structure composed of calcium phosphate-based, always mixed with hyaluronate, cellulose, and collagen [19]. Hydroxyapatite (HA) and the β form of its mixtures are the most synthetic calcium-based material to produce pastes [20]. The result of using these glues is to help proper regeneration of the bones. The regenerative forms of healing do not require constant support of osteoconductive abilities, for instance, removing the cyst from the mandible or maxillae, furcation defects, or other deep defects in the bone [21]. Permanent bone preservation in osteoporosis and bone tumors is a hallmark factor for stabilizing the size of organs. Then the researchers try to assemble the pastes materials. Xenogeneic pasting materials, including Cerabone and Neo-Oss, have been proved to contribute to this remodeling outline as they have been observed within their implantation areas several years after implantation. These glue materials maybe lead to a few inflammations and can retain the water as a bulk-forming laxative. We showed better-improved new bone regeneration in both material groups, suggesting proper osteoconduction. TCP and HA are xenogeneic materials that have already been studied and approved in various studies [4, 22, 23].

Shoaib (2021) reported that 65 ± 5 nm magnesium-doped mesoporous bioactive glass nanoparticles (Mg-MBG NPs) could load variable amounts of the drug. They showed a maximum cumulative release of 89% at a pH of 6.4 with no significant cytotoxicity in normal human fibroblast. Thus biocompatible Mg-MBG with low cytotoxicity and sustained drug release was a safe biomaterial [24]. Saifur Rahman 2020 showed that a controlled drug delivery system is a method for modeling bone regeneration and drug delivery for cancer treatment by chemotherapy. The release of doxorubicin could be controlled by the ability of the silver bioactive glass nanoparticles [25].

Shoaib (2017) proved that mesoporous bioactive glass could activate osteoblast activation and bone mineralization. This finding was shown by alkaline phosphatase activity and osteocalcin formation [26]. Mohseni et al. in 2017 revealed that the nanocomposite composed of the TCP/collagen could increase the volume and the rapidity of bone formation in the defect site better than HA composite operation after 30 and 45 days [4]. We found that the TCP/TiO2 nanocomposite could improve primary and lamellar bone formation in the defect site better than HA composite and autograft bone after 30, 45, and 60 days.

**Fig. 6:**
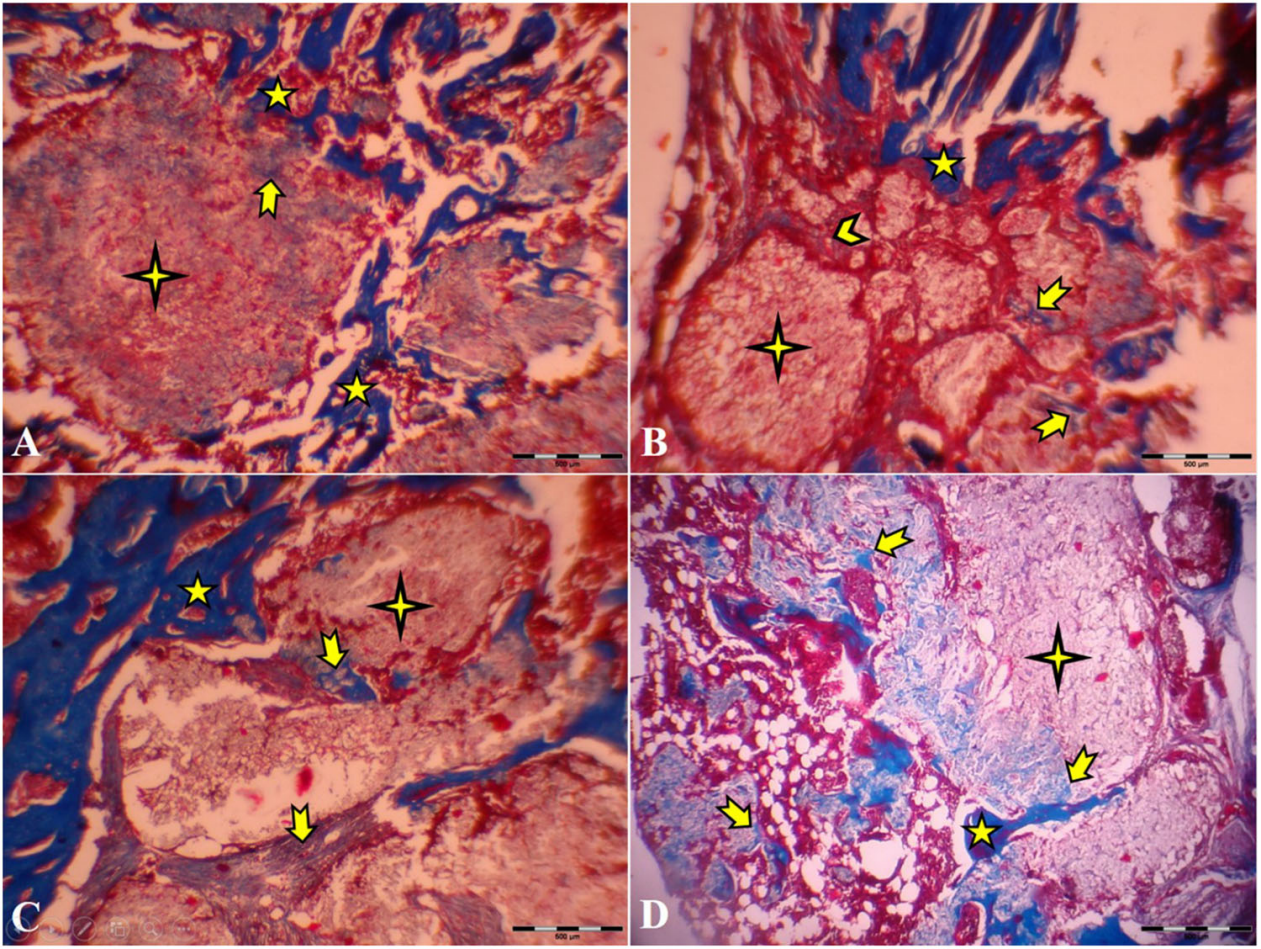
Microscopic section from the healing site of defect on the 30th day postoperation. **A** Control group developed scanty new bone around the granulation tissue. **B** The autograft group shows remains of the necrotic bone and more new bone formation. **C** HA that presents new bone formation. **D**: TCP-TiO2 materials in the center of the defect. The most developed new bone formation is shown in the TCP-TiO2 group (Trichrome × 40). star: remain previous bone; notched arrow: new bone formation; chevron: necrotic bone; 4-point star: granulation tissue

**Fig. 7:**
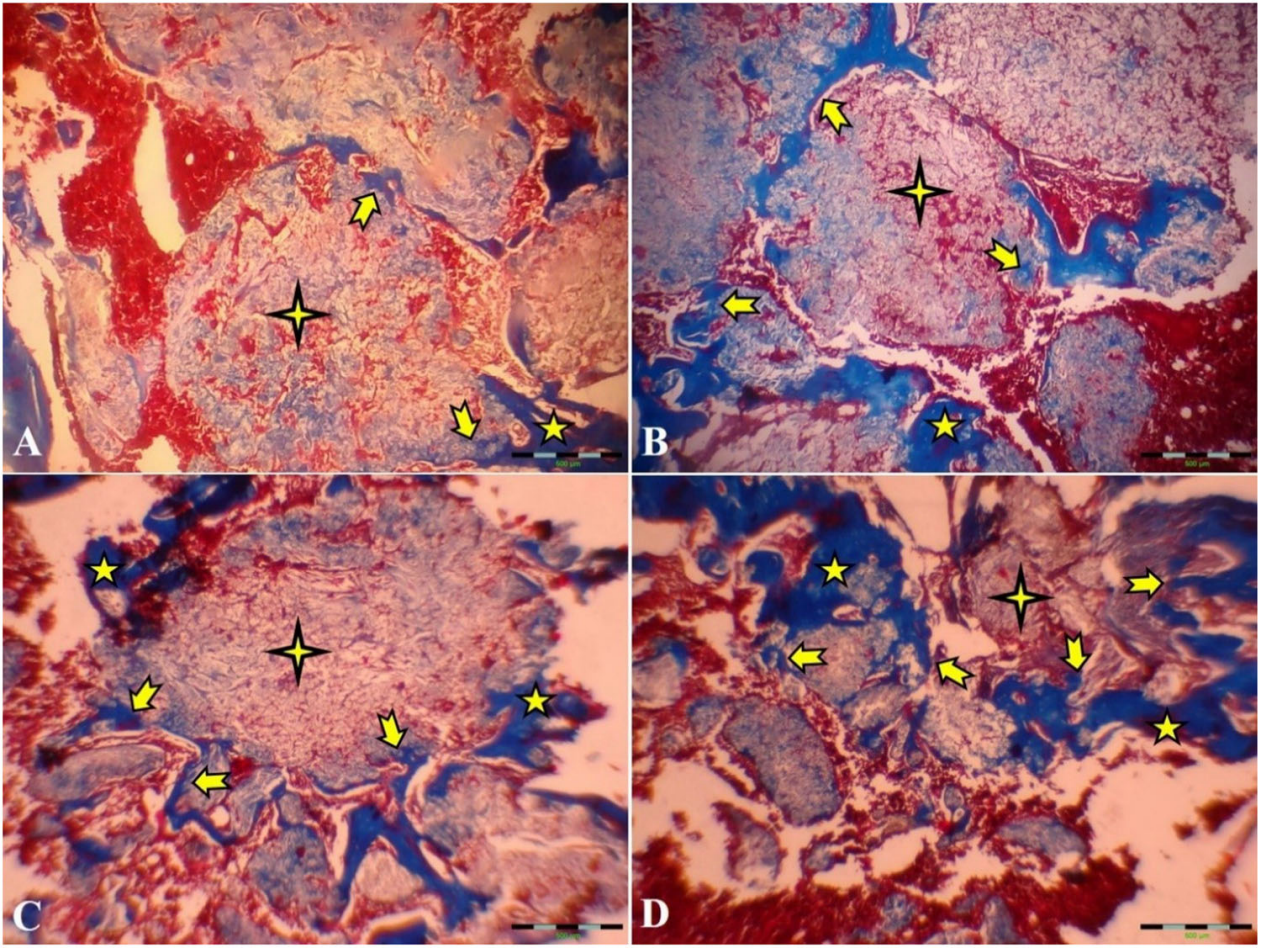
Microscopic section from the healing site of defect on the 45th day postoperation. **A** Control group few lamellar bones around the defective area. **B** The autograft group shows more lamellar bone formation than the control group. **C** HA group had a well-developed lamellar bone around the defect. **D** TCP-TiO2 group induced the most lamellar bone in the center of the defect (Trichrome × 40). star: remain previous bone; notched arrow: lamellar bone formation; 4-point star: center of the defect

According to our 45th- and 60th-day study, the primary new bone formation rate in the autograft and HA groups was significantly higher than in the control group. The primary new bone formation in both autograft and HA groups was lower than in the TCP-TiO2 group. This fact showed that the femur implantation site improved because of bone regeneration from the osteoconductive behavior of the TCP-TiO2. The results of the histopathological analysis on day 15 after surgery showed that the added TCP-TiO2 improved the new bone formation, similar to the bone autograft group. There was more leukocyte infiltration in the control group than in the others (Fig. 5A–D). This inflammation was against the findings from other researchers who had shown that adding pastes materials started more leukocyte infiltration [27]. The granulation tissue was in the center of all the defects on days 15 and 30 after surgery. The formation of new bone started more initially in the TCP-TiO2 group and autograft than in HA and control groups on day 30 after postoperation. There was no union structure still on the 30th day. The inflammatory reaction was reduced even in the control group. This analysis showed that none of the materials or autografts induced significant differences in improving compact cortical bone formation until the 30th day (Fig. 6A–D). Even because of the immune responses to biomaterials, the results show that the xenogeneic TCP-TiO2 did not appear to provoke an inflammatory response compared to the control group [28]. The healing of the defect site of the control group on day 45 postoperation presented new bone formation. The defect site of the HA-treated group on day 45th postoperation was sieged from around with the limited lamellar bone. While the healing area of the autograft and the nanocomposite TCP-TiO2 treated group on day 45 showed more lamellar bone development (Fig. 7A–D).

**Fig. 8:**
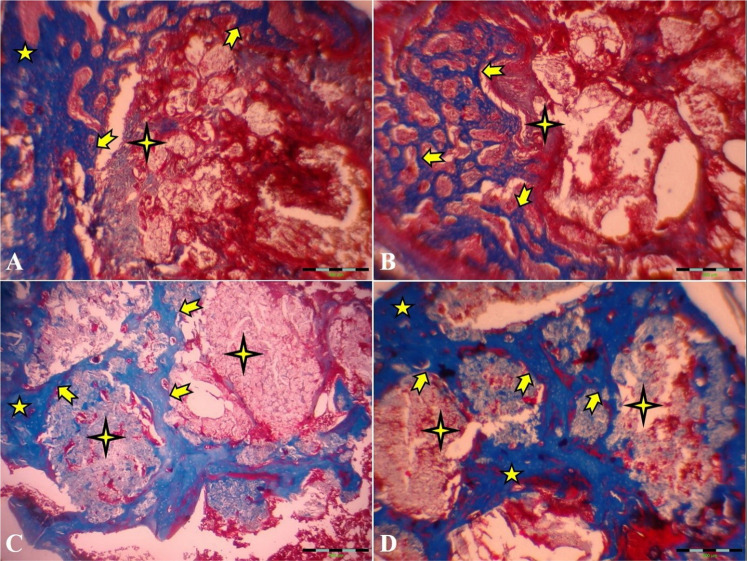
Microscopic section from the healing site of defect on the 60th day postoperation. **A** Thick lamellar bone was formed in the defective area in the control group. **B** The autograft group shows more diffuse thick and thin lamellar bone formation than the control group. **C** In the HA group, nearly half of the defective areas were filled by lamellar bone. **D** TCP-TiO2 group has the lamellar bone in more than half of the area of the defect (Trichrome × 40). star: remain previous bone; notched arrow: lamellar bone formation; 4-point star: center of the defect

Eventually, advanced lamellar bone development and remodeling of the spongy bone were seen on day 60 postoperation in all groups. The TCP-TiO2 group was the best improvement significantly compared to the others. The quantity of lamellar bone was significantly higher in the nanocomposite TCP-TiO2 treated group than in the HA and autograft-treated group (Fig. 8A–D). On 60 days post-surgery, the compact cortical bone was well-formed in all groups. The significant difference between the TCP-TiO2 and the control group was higher than the similar differences were the autograft and HA-treated groups. Finally, the measurements of the marrow deployment showed a weak density of the precursor cells in all study groups. The analysis of the cortical bone development did not reveal any significant differences within the different time points except on day 60 postoperation. These data also conclude that either the autograft or the HA group provoked bone formation. These reactions are consistent, as previously shown by the other research components [2, 4, 15, 29].
